# Comparative Transcriptome Analysis Reveals the Mechanism Associated With Dynamic Changes in Meat Quality of the Longissimus Thoracis Muscle in Tibetan Sheep at Different Growth Stages

**DOI:** 10.3389/fvets.2022.926725

**Published:** 2022-07-06

**Authors:** Yuliang Wen, Shaobin Li, Gaoliang Bao, Jiqing Wang, Xiu Liu, Jiang Hu, Fangfang Zhao, Zhidong Zhao, Bingang Shi, Yuzhu Luo

**Affiliations:** Gansu Key Laboratory of Herbivorous Animal Biotechnology, Faculty of Animal Science and Technology, Gansu Agricultural University, Lanzhou, China

**Keywords:** RNA sequencing, Tibetan sheep, muscle tenderness, different growth stages, muscle development, meat quality

## Abstract

Tibetan sheep are mainly distributed in the Qinghai–Tibet Plateau. Its meat is not only essential for the local people but also preferred by the non-inhabitant of this plateau also. To investigate the salient development features and molecular mechanism of the meat difference of LT muscle caused by different growth stages in Tibetan sheep, the carcass performance, meat quality, and comparative transcriptome analysis were performed for investigating the potential molecular mechanism of the meat quality difference of the LT muscle caused by four growth stages [4-months old (4 months), 1.5-years old (1.5 years), 3.5-years old (3.5 years), and 6-years old (6 years)] in the Tibetan sheep. The shear force increased with the increase of age (*p* < 0.05) while the intramuscular fat (IMF) was the highest at 1.5 y. The AMPK signaling pathway was significantly enriched in the four comparative groups. The weighted gene co-expression network analysis (WGCNA) results showed that the hub genes *P4HA2, FBXL4*, and *PPARA* were identified to regulate the meat quality. In summary, 1.5 years was found to be the most suitable slaughter age of the Tibetan sheep which ensured better meat tenderness and higher IMF content. Moreover, the genes *LIPE, LEP, ADIPOQ, SCD*, and *FASN* may regulate the transformation of the muscle fiber types through the AMPK signaling pathway, further affecting the meat quality.

## Introduction

Meat quality traits constitute important economic traits of livestock. The molecular mechanism of skeletal muscle growth and muscle tenderness is conducive to improving livestock performance and meat quality. Tibetan sheep (*Ovis aries*) are mainly distributed on the Qinghai–Tibet Plateau above 3,000-m altitude. The sheep is wholly adapted to the special ecological environment with extreme conditions, such as lower temperature, thinner oxygen level, and stronger ultraviolet rays. The sheep mainly produces meat and is loved by all the consumers in Northwest China for its unique flavor and palatability, which is influenced by the natural environment (1). However, it remains elusive whether this living environment is related to the type of muscle energy metabolism in Tibetan sheep. The number of skeletal muscle fibers is constant in mammals after birth (2, 3). The growth and development of muscle fiber mainly includes the increase of the muscle fiber diameter and the muscle fiber types transition after birth (4). Moreover, there are real differences in the structure, function, and metabolism between the different muscle fibers types, which further influencing the meat quality. Hence, it is crucial to investigate the skeletal muscles growth and development in livestock after birth for the meat traits. Tenderness is an important index to evaluate the meat quality, and also influences the consumers' intention for purchase and its market acceptance (5). Saccà et al. (6) have identified the goat meat tenderness decreased with the increase of age. Nevertheless, it remains elusive whether muscle development influences meat tenderness in livestock after birth growth.

Currently, there have been a lot of studies on the mechanism of skeletal muscle transcriptome and proteome in the species such as cattle (7, 8), sheep (9, 10), and goats (11). The previous studies have demonstrated the important role of the related genes and signaling pathway in muscle growth and development, including the myogenic transcription factor Pax3 and Pax7 (12); the myogenic regulatory factors Myf5, MyoD, and myogenin (13); the fibroblast growth factor (FGF); and transforming growth factor β (TGF-β). The skeletal muscle development and differentiation were regulated through these regulatory factors and Wnt, AMPK, MAPK, and PI3K-Akt signaling pathways (14). The skeletal muscle is composed of a variety of functionally diverse fiber types, there are mainly four types of muscle fibers in the adult mammalian skeletal muscle, including MyHC I, MyHC IIa, MyHC IIx, and MyHC IIb.

The previous studies have identified that the different types of muscle fibers to influence many meat quality traits such as the water-holding capacity (WHC), tenderness, color, juiciness, and flavor (15). Liu et al. (16) demonstrated CaN/NFAT as an important signaling pathway, regulating the transformation of the chicken muscle fiber types. Zhu et al. (17) found MYH6 and Ca^2+^ signaling pathways to regulate the muscle fiber types of skeletal muscle in large white pigs. In addition, the previous studies have also found that increasing the AMPK activity can change the proportion of fiber types in pork (18). However, the molecular mechanism of the LT muscle fiber types transition in the Tibetan sheep at different growth stages, and its influence on the meat tenderness remains largely unknown.

There is still a lack of systematic research on the regulation of changes in the muscle fiber types especially during the growth and development of the skeletal muscle of the animals after birth. In the few years, the next generation sequencing technology and bioinformatics methods have developed rapidly, like the RNA-seq, the molecular mechanism of muscle tenderness changes at different growth stages in Tibetan sheep was explored simply and effectively. The comparative transcriptome analysis of the LT muscle was used for identifying the regulatory genes related to meat tenderness and meat quality at different growth stages in Tibetan sheep. Furthermore, the functions and signaling pathways of DEGs were investigated. The WGCNA regulatory network was further used to analyze the hub genes related to meat quality.

## Materials and Methods

### Ethics Statement

The animal study was reviewed and approved by the Faculty Animal Policy and Welfare Committee of Gansu Agricultural University (Ethic approval file No. GSAU-Eth-AST-2021-001).

### Animals and Muscle Sampling

A total of 16 healthy female Tibetan sheep were selected from the same flock of the Haiyan County, Qinghai Province, China, including 4 months (*n* = 4), 1.5 years (*n* = 4), 3.5 years (*n* = 4), and 6 years (*n* = 4). For the detailed samples collection process, see our previous study (19). Then, the initial weight, hot carcass weight, and dressing percentage were recorded and the meat quality were determined. The sample collection and processing for RNA extraction were carried out according to the results of our previous study (19).

### Meat Quality Measurements

The shear force was measured according to the methods as described by Honikel (1998) (20). The process included the meat samples from Tibetan sheep for 48 h after slaughter were cooked in the cooking bags until the internal temperature was maintained at 75°C. Then taking out the meat samples and cooling them, using a sampler with a diameter of 1.27 cm to take meat slices and measuring shear force by a shearing device (C-LM3B, Runhu Instrument Co., Ltd., Guangzhou, China). The intramuscular fat (IMF) and crude protein content in mutton was determined according to the methods as described by AOAC (2007) (21). The experiment was repeated 3 times in each group (22).

### RNA Extraction and Sequencing

The total RNA was extracted according to the methods as described by Bao et al. (23). After the total RNA was extracted, Illumina TruSeq TM RNA (Illumina, USA) kit was used to construct cDNA libraries for RNA sequencing and sequenced using Illumina Novaseq6000 (or other platforms) by Gene Denovo Biotechnology Co. (Guangzhou, China).

### Raw Data Cleaning and Transcriptome

The raw data cleaning was filtered by fastp (v0.18.0) (24). The detailed process can refer to our previous study (23). Bowtie2 (v2.2.8) (25) was used to identify ribosomal RNA and were removed. The remaining clean reads were mapped the sheep *Oar*_v1.0 reference genome using HISAT2 (26) (v2.1.0). The software Stringtie (v1.3.4) (27, 28) was used to reconstruct the transcripts. All software used default parameters.

### Differentially Expressed Genes Analysis

The fragment per kilobase of transcript per million mapped reads (FPKM) was used to calculate the expression levels of mRNA using RSEM (29). The DEGs were analyzed by DESeq2 (30) and edgeR (31). The false discovery rate (FDR) < 0.05 and |log_2_(Fold Change)| > 1 were used to identify DEGs.

### Gene Ontology (GO) Enrichment and Kyoto Encyclopedia of Genes and Genomes Pathway Analysis of the DEGs

In this study, DAVID (http://david.abcc.ncifcrf.gov/) online analysis software was used for GO function annotation and KEGG pathway enrichment analysis (32).

### Weighted Gene Co-Expression Network Analysis

The WGCNA was performed using the WGCNA R software (33) for constructing a co-expression network. The top 50% of genes with the largest variation for WGCNA were selected after threshold screening while calculating degree of variation in the expression level of each gene between samples. For power processing on the original scaled relation matrix, β = 8 was finally selected, and the unscaled adjacency matrix was generated. In addition, the correlation between the modules and meat quality traits of the LT muscle of Tibetan sheep were investigated using Pearson's correlation.

### Gene Co-Expression Network

To analyze the selected module gene, The Search Tool for the Retrieval of Interacting Genes (STRING) database (v11.5) was used to construct the network. The top-10 connectivity within the module genes was screened for constructing the network, and it was displayed by the Cytoscape software (34).

### Gene Expression Analysis With RT-qPCR

The real-time quantitative polymerase chain reaction (RT-qPCR) was used to verify the authenticity of the transcriptome results according the methods as described by Wen et al. (35). The primers were designed by Primer (v5.0), and listed in Supplementary Table 1. *GAPDH* was used as a reference gene for calculating the relative expression according to 2^−Δ*ΔCt*^ method (36).

### Statistical Analysis

Statistical analyses included the ANOVA, followed by Duncan's multiple range test for multiple comparisons of the difference of carcass quality and meat quality in SPSS 20.0 software (SPSS, Armonk, NY, USA). All data in this study were presented as mean ± standard error (SEM), *p* < 0.05 and different lowercase letters means that the difference was significant.

## Results

### Carcass Performance and Meat Quality of the Tibetan Sheep

As shown in Table 1, the muscle development associated with live weight and carcass weights was found to increase with the increase of age. The live weight was increased from 4 months to 3.5 years (*p* < 0.05). The hot carcass weight was increased from 4 months to 3.5 years (*p* < 0.05), while the weight was decreased from 3.5 years to 6 years (*p* < 0.05). The dressing percentage was largest at 1.5 years, and then decreased with the increase of age (*p* < 0.05). The IMF content was increased from 4 months to 1.5 years (*p* < 0.05), and then decreased with the increase of age. The shear force was increased with the increase of age (*p* < 0.05). The crude protein was increased with the increase of age, while there was no significant difference between the 3.5 years and 6 years sheep.

**Table 1:** Carcass quality and meat quality of Tibetan sheep.

**Parameters**	**4 months**	**1.5 years**	**3.5 years**	**6 years**	***p***
Live weight (kg)	14.51 ± 0.50^c^	26.42 ± 1.59^b^	34.00 ± 1.90^a^	33.75 ± 2.62^a^	<0.001
Hot carcass weight (kg)	6.45 ± 0.24^c^	12.77 ± 1.20^b^	15.61 ± 1.08^a^	13.53 ± 1.22^b^	<0.001
Dressing percentage (%)	44.43 ± 1.12^b^	48.26 ± 1.82^a^	45.88 ± 0.71^b^	40.08 ± 1.20^c^	<0.001
IMF (%)	1.60 ± 0.32^b^	2.46 ± 0.49^a^	2.36 ± 0.39^a^	1.63 ± 0.26^b^	<0.001
Shear force (*N*)	26.87 ± 3.17^d^	42.86 ± 3.64^c^	50.77 ± 4.18^b^	59.96 ± 3.85^a^	<0.001
Protein (%)	17.39 ± 0.14^c^	19.90 ± 0.18^b^	21.50 ± 0.41^a^	21.82 ± 0.16^a^	<0.001

*Date shown are means ± SEM and p < 0.05 and different lowercase letters indicate the difference was significant*.

### Summary of the RNA-Seq Data

Total 16 libraries were constructed in this study. As shown in Supplementary Table 2, 91,167,071, 95,150,638, 90,313,673, and 87,472,429 average raw reads were generated from four growth stages respectively, the correlation coefficient between 4 months, 1.5 years, 3.5 years, and 6 years samples of Tibetan sheep were 0.983, 0.890, 0.962, and 0.990, respectively. Of the remaining clean reads, there was more than an average of 82 million (92.04 %) mapped to the reference genome.

The criterion of FPKM > 0.01 was used for identifying the potentially expressed genes (37). A total of 9,912, 10,011, 10,048, and 9,788 expressed genes were identified in the LT muscle tissues from the four growth stages of Tibetan sheep, respectively, and 9,241 genes were co-expressed in four ages.

### Analysis of DEGs

**Figure 1:**
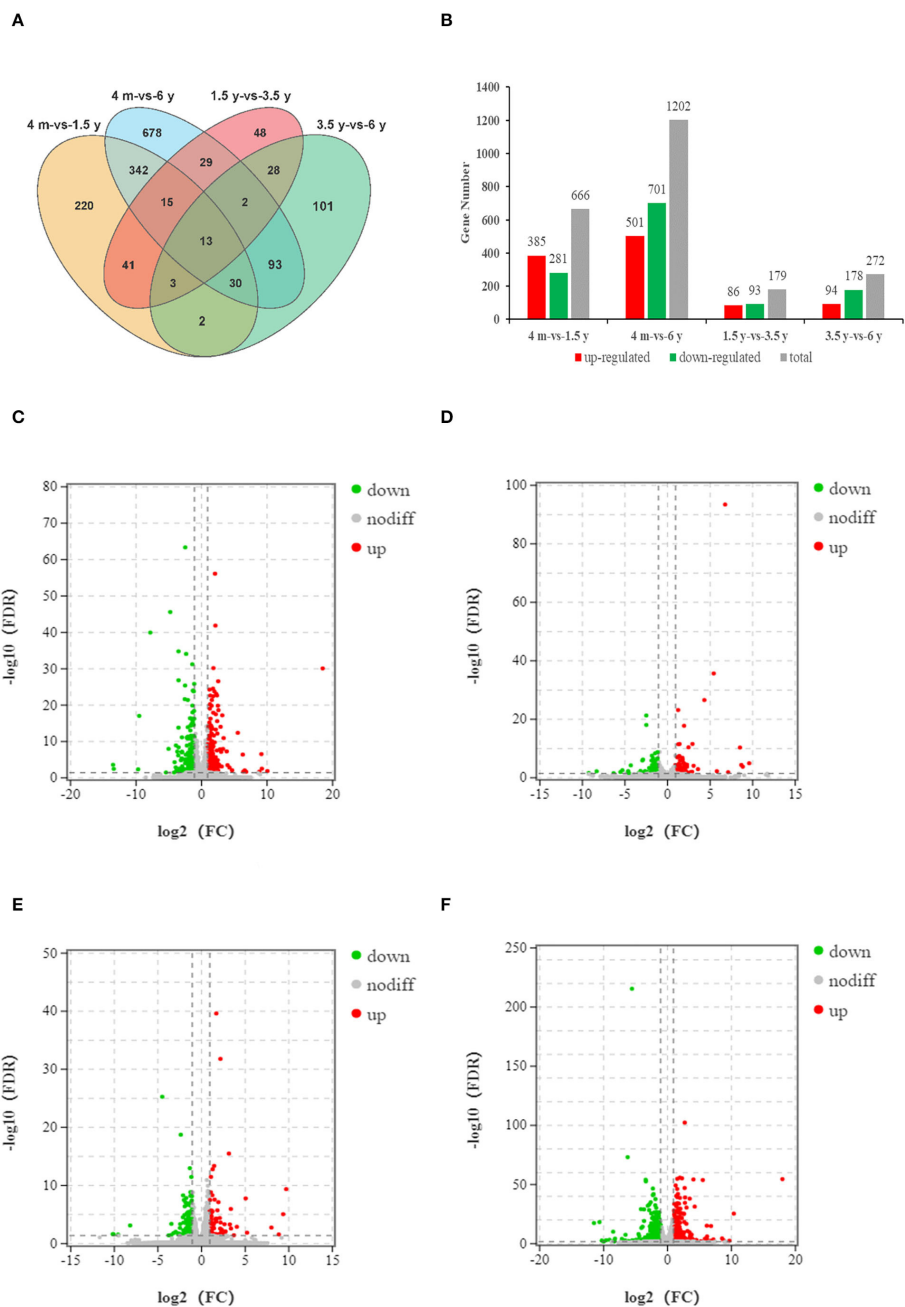
Analysis of the DEGs of four groups. **(A)** The Venn diagram of DEGs. **(B)** Histogram of DEGs of four comparative groups. **(C–F)** Volcano diagrams of DEGs in group of 4 months *vs*. 1.5 years, 1.5 years *vs*. 3.5 years, 3.5 years *vs*. 6 years, and 4 months *vs*. 6 years, respectively. The abscissa represents the logarithm of the fold change of DEGs between the two groups, and the ordinate represents the negative log_10_ value of the FDR of the DEGs between the two groups. A point in the volcano represents a gene, red represents up-regulated genes, green represents down-regulated genes, and black represents non-differentially expressed genes.

To analyze the timing of muscle growth and development in the Tibetan sheep, the DEGs of the contiguous period transcriptome comparative groups (4 months *vs*. 1.5 years, 1.5 years *vs*. 3.5 years, 3.5 years *vs*. 6 years, and 4 months *vs*. 6 years) were identified, each group was identified with 220, 48, 101, and 678 unique DEGs, respectively (Figure 1A; Supplementary Table 3). Overall, there were 666 (385 up-regulated; 281 down-regulated), 179 (86 up-regulated; 93 down-regulated), 272 (94 up-regulated; 178 down-regulated), and 1,202 (501 up-regulated; 701 down-regulated) DEGs were identified in 4 months *vs*. 1.5 years, 1.5 years *vs*. 3.5 years, 3.5 years *vs*. 6 years and 4 months *vs*. 6 years groups, respectively (Figures 1B–F).

### GO and KEGG Enrichment Analysis of DEGs

**Figure 2:**
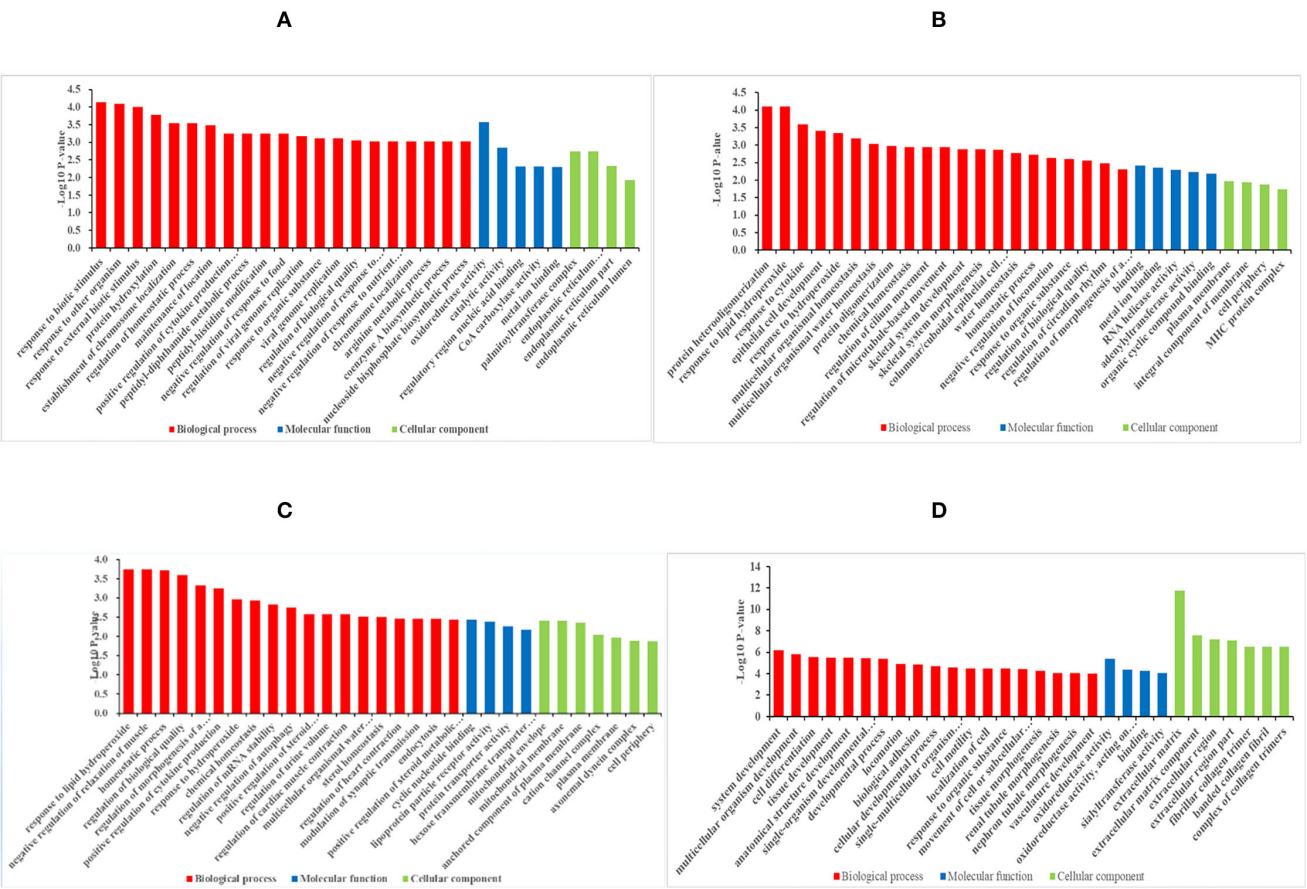
Histogram diagram of DEGs in GO enrichment. **(A–D)** Histogram diagram of GO enrichment results of DEGs in group of 4 months *vs*. 1.5 years, 1.5 years *vs*. 3.5 years, 3.5 years *vs*. 6 years, and 4 months *vs*. 6 years, respectively.

The GO enrichment analysis can assist in understanding the function of DEGs between each group more comprehensively, similar to most studies, the DEGs were significantly enriched into the following three main GO categories: Biological processes (BP), molecular functions (MF), and cellular components (CC). Based on the GO significant enrichment analysis (*P* < 0.05), and the annotation of the GO database 666 DEGs of 4 months *vs*. 1.5 years group were found to be significantly enriched to 226 BP, 51MF, and 11 CC (Supplementary Table 4). Most of the DEGs were mainly divided into the functional groups, including those related to biosynthesis, nutrient metabolism, and development (Figure 2A). The GO term involved in the function of organ growth (GO: 0035265), endochondral bone growth (GO: 0003416), and regulation of lipid metabolic process (GO: 0019216). Among these terms, the most significantly enriched GO term constituted a response to the biotic stimulus (GO: 0009607) (*p* = 7.39E-5). Also, many DEGs were involved in the coenzyme A metabolic process (GO: 0015936), where the number of genes enriched in the term of binding (GO: 0005488) was the most significant. The organs and bones were speculated to develop first in this stage, and the energy metabolism was relatively vigorous. The IMF content was found to increase significantly from the age of 4 months to 1.5 years, possibly related to the active fatty acid metabolism. The crude protein content also increased significantly in this stage, and was related to amino acid metabolism.

Based on the significant enrichment analysis (*p* < 0.05), the DEGs in the 1.5 years *vs*. 3.5 years group were found to be significantly enriched in 146 BP, 33 MF, and 10 CC (Supplementary Table 4). The GO term was involved in the function of synthesis, cell differentiation, and metabolic regulation (Figure 2B), including regulation of fatty acid metabolic process (GO: 0019217) and epithelial cell differentiation (GO: 0030855). Among these terms, protein heterooligomerization (GO:0051291) (*p* = 7.77E-5) was the most significantly enriched GO term. The protein content increased during this growth stage due to the functions linked with protein synthesis. Although DEGs were also enriched in the functions such as fat synthesis and fatty acid metabolism, the IMF content decreased compared to that in the 1.5-years old, with no significant difference. This might be accountable to the lipids deposited in the subcutaneous fat or other lipids for providing energy.

Based on the significant enrichment analysis (*p* < 0.05) the DEGs in the 3.5 years *vs*. 6 years group were significantly enriched to 230 BP, 38 MF, and 26 CC (Supplementary Table 4). The GO term involved in the function of metabolism and regulation (Figure 2C) included negative regulation of relaxation of muscle (GO: 1901078), regulation of striated muscle contraction (GO: 0006942), and positive regulation of lipid biosynthetic process (GO: 0046889). Also, many DEGs were involved in regulating of autophagy (GO: 0010506). Among these terms, a response to lipid hydroperoxide (GO:0006982) (*p* = 0.00018) was the most significantly enriched GO term. It showed the difference in the contractility of different types of muscle fibers, and the IMF was found to significantly decrease due to negative regulation of the lipid biosynthesis.

Based on the significant enrichment analysis (*p* < 0.05), the DEGs in the 4 months *vs*. 6 years group were significantly enriched to 337 BP, 46 MF, and 31 CC (Supplementary Table 4). The GO term involved in the function of development, differentiation and metabolism (Figure 2D). The most significantly enriched GO term was the extracellular matrix (GO: 0031012) (*p* = 1.84E-12). The DEGs were mainly enriched in the development, differentiation, and metabolism of the skeletal muscle and other tissues, it was mainly enriched in the process of maturation to aging of the body.

**Figure 3:**
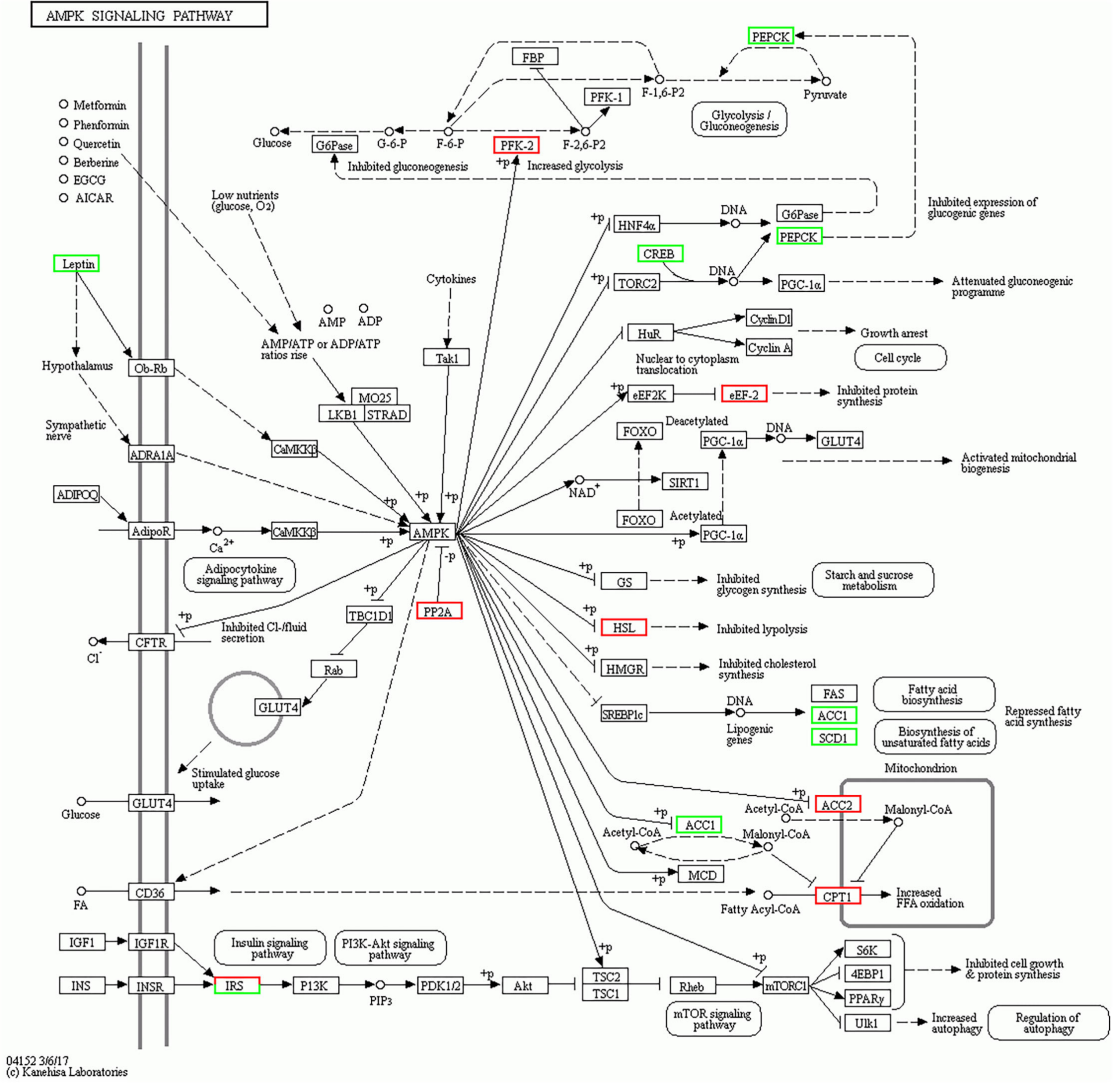
AMPK signaling pathway in the 4 months *vs*. 1.5 years group. The white square represents a gene or protein, the red square is the up-regulated genes in the pathway at this time, the green square is the down-regulated genes in the pathway at this time.

The KEGG pathway was used for further analyzing the potential functional signaling pathways of DEGs (Table 2). The results showed that the DEGs in the 4 months *vs*. 1.5 years group were significantly enriched in the AMPK signaling pathway (*p* = 0.00006), and there were 14 DEGs annotated in this pathway, which were closely related to the connection of the muscle cells (Figure 3). The lipid metabolism related pathway, including PPARA, SCD, and APOA1 were significantly enriched in the PPAR signaling pathway (*p* < 0.05), and a total of 10 DEGs were annotated. In addition, the adipocytokine, and antigen processing signaling pathway, were also significantly enriched (*p* < 0.05).

**Table 2:** The significant KEGG enrichment results of DEGs.

**KEGG ID**	**Description**	**Pathway**	***p***	***Q***	**Involved DEGs**
**4 months** ***vs***. **1.5 years**					
ko04152	Signal transduction	AMPK signaling pathway	0.000060	0.005772	LIPE; PCK1; IRS2; PFKFB2; LEP; EEF2; ACC1
ko03320	Endocrine system	PPAR signaling pathway	0.000547	0.039242	PPARA; SCD; APOA1
ko04920	Endocrine system	Adipocytokine signaling pathway	0.002160	0.101659	CPT1A; irs1; PRKCQ; ACACB
ko04612	Immune system	Antigen processing and presentation	0.002526	0.101659	HSPA1B; CALR; HLA-A
ko04145	Transport and catabolism	Phagosome	0.004407	0.129284	CALR; VAMP3
**1.5 years** ***vs***. **3.5 years**					
ko00480	Metabolism of other amino acids	Glutathione metabolism	0.003545	0.059359	Gstm5; MGST1; GSTM1
ko04514	Signaling molecules and interaction	Cell adhesion molecules (CAMs)	0.005995	0.075117	VCAM1; CDH1
ko04371	Signal transduction	Apelin signaling pathway	0.012401	0.098774	PRKAG2; EGR1; MYLK4
ko04910	Endocrine system	Insulin signaling pathway	0.017059	0.121179	SOCS3; PIK3R3
ko04152	Signal transduction	AMPK signaling pathway	0.038030	0.226226	ADIPOQ;
**3.5 years** ***vs***. **6 years**					
ko04514	Signaling molecules and interaction	Cell adhesion molecules (CAMs)	0.001215	0.274657	CLDN4; VCAM1; CNTN2
ko04931	Endocrine and metabolic diseases	Insulin resistance	0.002760	0.311832	CREB5; RPS6KA6
ko04973	Digestive system	Carbohydrate digestion and absorption	0.021524	0.745368	HK2; ATP1B3
ko04928	Endocrine system	Parathyroid hormone synthesis, secretion and action	0.045204	0.745368	PDE4D; CDKN1A
ko04152	Signal transduction	AMPK signaling pathway	0.079958	0.745368	SLC2A4; CREB5
**4 months** ***vs***. **6 years**					
ko04360	Development	Axon guidance	0.000139	0.041303	RND1; ROCK2; PAK4; NTN3
ko04512	Signaling molecules and interaction	ECM-receptor interaction	0.000269	0.041303	SDC1; LAMA5; TNC
ko04510	Cellular community	Focal adhesion	0.000491	0.050271	MAPK10
ko04068	Signal transduction	FoxO signaling pathway	0.004299	0.119973	IGF1R; FoxO6
ko04152	Signal transduction	AMPK signaling pathway	0.005885	0.130668	IGF1R; FASN

The KEGG enrichment results were shown in Supplementary Table 5. Specifically, the DEGs in the 1.5 years *vs*. 3.5 years group were also significantly enriched (*p* = 0.038) in the AMPK signaling pathway and *ADIPOQ* was annotated (Table 2; Supplementary Figure 1A). *Gstm5, MGST1*, and *GSTM1* were significantly enriched (*p* < 0.05) in glutathione metabolism, and was related to the protein synthesis, and there were 4 DEGs annotated in this pathway. In addition, the cell adhesion molecules (CAMs), Apelin signaling pathway and Insulin signaling pathway were significantly enriched (*p* < 0.05).

The DEGs in the 3.5 years *vs*. 6 years group were significantly enriched (*p* = 0.001215) in the cell adhesion molecules (CAMs), which were related to cell recognition, signal transduction, activated proliferation, and differentiation. A total of eight DEGs were annotated in the above pathway. The DEGs in the 3.5 years *vs*.6 years group were also enriched in the AMPK signaling pathway (*p* = 0.079), *SLC2A4*, and *CREB5* were enriched in this signaling pathway (Table 2; Supplementary Figure 1B). In addition, insulin resistance, carbohydrate digestion, absorption, and action were significantly enriched (*p* < 0.05).

There were 24 DEGs in the 4 months *vs*. 6 years group were found to be significantly enriched in the axon guidance (*p* = 0.0001393), while 15 DEGs were found to be significantly enriched in the AMPK signaling pathway (*p* < 0.05) (Table 2; Supplementary Figure 1C).

### Co-Expression Module of WGCNA

**Figure 4:**
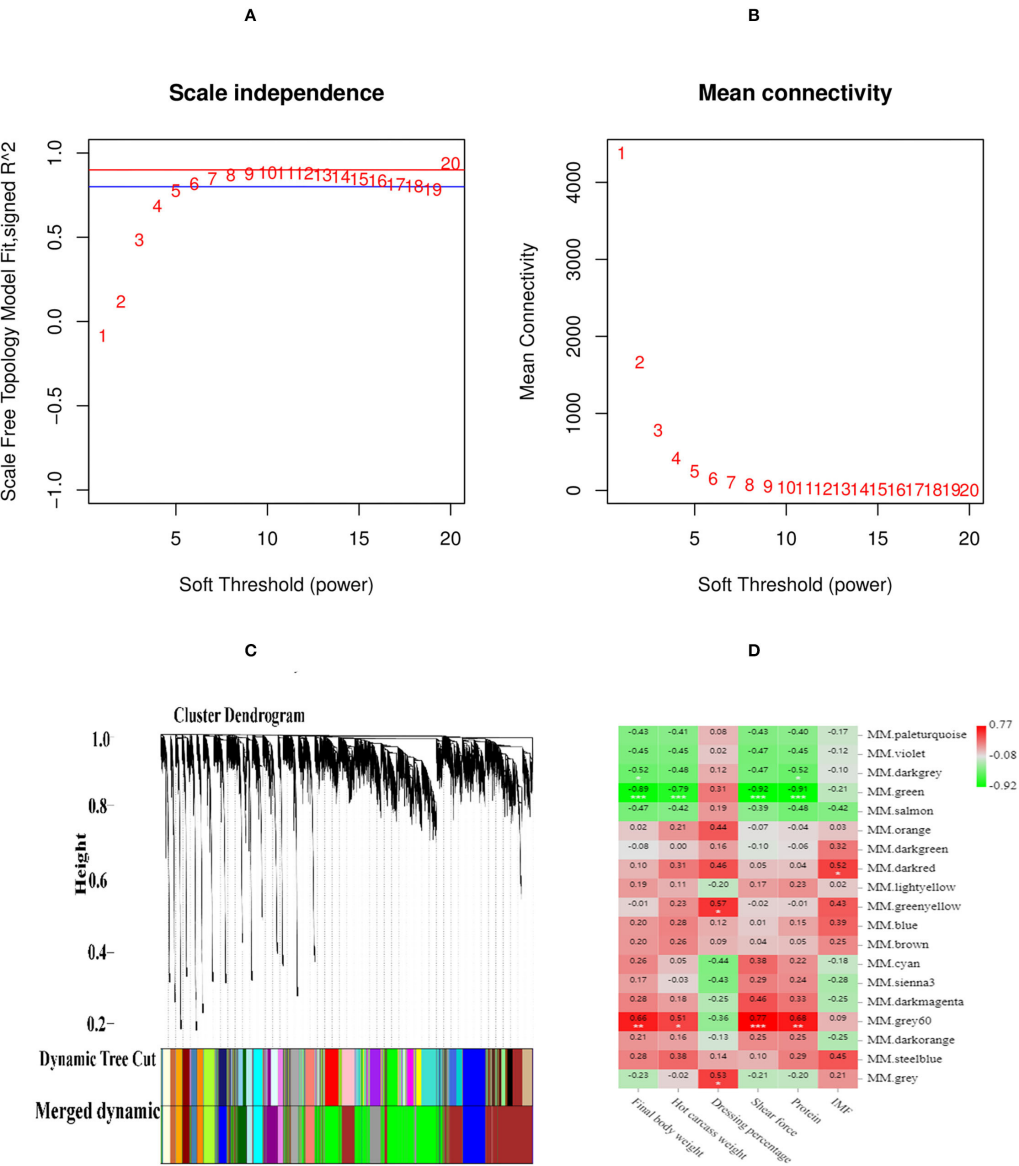
The scale-free fit index and mean connectivity of WGCNA. **(A)** Abscissa represents the soft threshold (power value, β). Ordinate represents the correlation coefficient *R*^2^. Blue horizontal line represents the correlation coefficient *R*^2^ = 0.8, red horizontal line represents the correlation coefficient *R*^2^ = 0.9. **(B)** Abscissa represents the soft threshold (power value, β), Ordinate represents the mean connectivity. **(C)** Clustering tree (dendrogram) defined by WGCNA representing the co-expression modules. Branches of the dendrogram correspond to modules labeled with different colors below the dendrogram. **(D)** Correlation between modules and meat quality and slaughter trait according to Pearson correlation.

The scale-free fit index and mean connectivity were calculated, selecting the soft-thresholding power of β = 8. The scale free *R*^2^ > 0.8, mean connectivity tends to zero revealed that the power of β = 8 to power processing could construct a scale-free network (Figures 4A,B). The weighted gene co-expression network analysis divided the DEGs of four groups into various modules. The dynamic shearing algorithm clustered and partitioned the genes, and the module feature vector of each module was calculated, merging the similar modules. As a result, 19 modules were determined (Figure 4C). The significant negative correlations were found between the MM.green and live weight (*r* = −0.89, *p* < 0.001), hot carcass weight (*r* = −0.79, *p* < 0.001), shear force (*r* = −0.92, *p* < 0.001), and crude protein (*r* = −0.91, *p* < 0.001). There were significant positive correlations found between the MM.gray 60 and shear force (*r* = 0.77, *p* < 0.001), live weight (*r* = 0.66, *p* < 0.01), and crude protein (*r* = 0.68, *p* < 0.01). There were significant positive correlations between the MM.dark-red and IMF (*r* = 0.52, *p* < 0.05) (Figure 4D).

### Identification of the Hub Gene and Construction of the Gene Co-Expression Network

**Figure 5:**
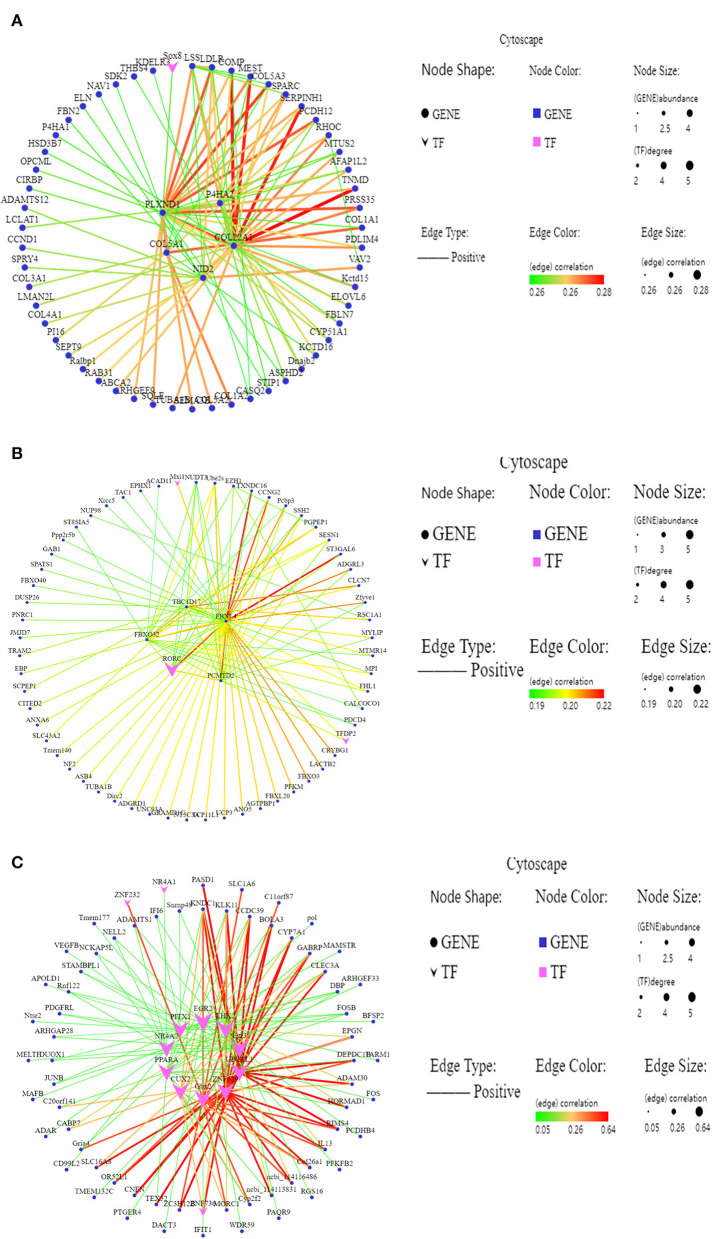
The gene co-expression regulatory interaction network. **(A–C)** Gene co-expression network of the MM.green module, MM.grey60 module, MM.dark-red module, respectively. Larger and darker colors in large lines in the corresponding network indicate genes with higher connectivity; vee represents transcription factors; ellipse represent other genes.

The hub genes usually refer to the genes with high connectivity within the module. The gene interaction network of MM.green module was constructed, and the top five genes (*P4HA2, PLXND1, COL22A1, COL5A1*, and *NID2*) and a transcription factor (Sox8) were identified as the hub genes (Figure 5A). *P4HA2* and *COL5A1* are related to collagen synthesis, *COL22A1* is related to skeletal muscle contraction, and the transcription factor Sox8 is involved in regulating embryonic development. The top four genes (*FBXL4, FBXO32, TBC1D17*, and *PCMTD2*) and a transcription factor (RORC) were identified with the highest degree of connectivity in the interaction network of MM. *grey60* module as hub genes (Figure 5B). *FBXL4* was found to play a critical role in the cell cycle, *FBXO32* was related to muscle atrophy, and *TBC1D17* is related to mitochondrial autophagy; RORC is a DNA binding transcription factor. The top four transcription factors (PPARA, ADAM30, UBTFL1, and EGR2) and a gene (*KNDC1*) were identified to possess the highest degree of connectivity in the interaction network of MM.dark-red module as hub genes (Figure 5C). Among these genes, PPARA was related to fat deposition, EGR2 was involved in fat metabolism, UBTFL1 was involved in early development and the origin of embryonic stem cells, ADAM30 was involved in muscle development.

### RT-qPCR Validation

**Figure 6:**
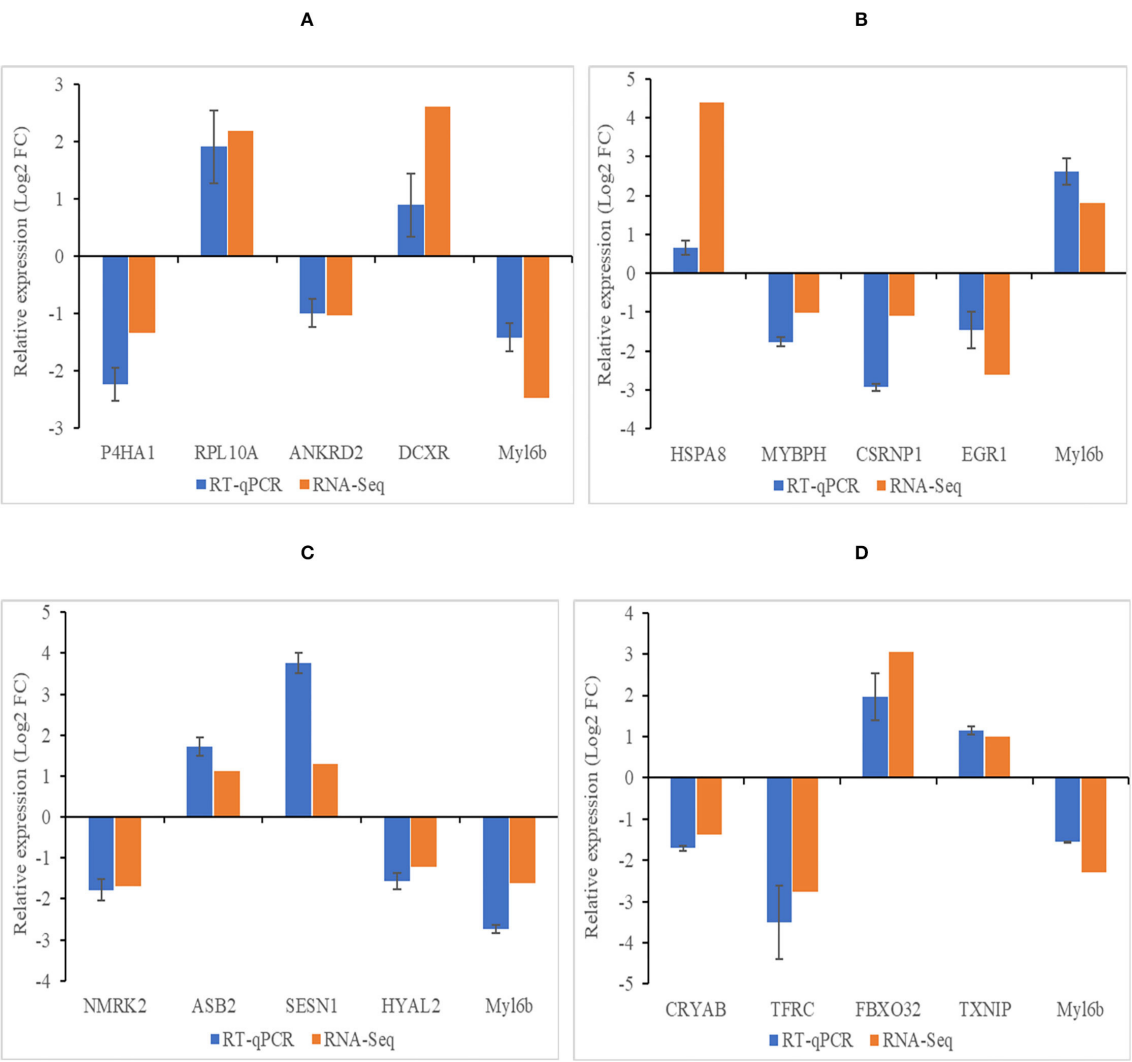
Verification results of DEGs by RT-qPCR. **(A–D)** Histogram diagram of validation results in group of 4 months *vs*. 1.5 years, 1.5 years *vs*. 3.5 years, 3.5 years *vs*. 6 years, and 4 months *vs*. 6 years, respectively. Abscissa represents genes in different compare group, ordinate represents the relative fold change. Data shown on figure are mean ± SEM.

There were 20 genes randomly selected for validation by RT-qPCR in different compare group, and the RT-qPCR results were consistent with the RNA-seq results (Figures 6A–D).

## Discussion

This study revealed the molecular mechanism of the difference in the meat yield, meat quality, and muscle tenderness of the Tibetan sheep at different growth stages. The meat tenderness mainly influences the consumers' intention for purchase as well as the key factor for its market acceptance (4). The IMF content critically influences the tenderness, juiciness, and flavor, and has a positive impact on meat tenderness (38). Similar to the study by Pascual–Alonso et al. (39), the increase in the sheer force with the increase of age (*p* < 0.05). In this study, was the highest in the 6 years Tibetan sheep meat. The IMF content increased from 4 months to 1.5 years, and then decreased and this was consistent with the result of Saccà (2019) study on the goat meat at different growth stages (5). In this study, the IMF content and dressing percentage of Tibetan sheep were the highest in 1.5 years; therefore, 1.5 years is the most suitable slaughter age for Tibetan sheep and yields better meat quality.

Transcriptome analysis has been applied in many applied research. This novel study has revealed the molecular mechanism of differences in Tibetan sheep meat tenderness at different ages using the RNA-seq approach. In this study, four samples were selected each group. However, based on the expression of the entire gene in any two samples, the correlation coefficient of every two samples were calculated, and the results showed the correlation coefficients of the Tibetan sheep to be above 0.983, 0.890, 0.962, and 0.990 at 4 months, 1.5 years, 3.5 years, and 6 years, respectively, Hence, the four sample sizes were found to satisfy the reliability of the RNA-seq results (11).

There are two stages of muscle growth and development in mammals, before and after birth. During the fetal period of development stage before birth, a group of mesenchymal cells located paraxial mesoderm of the embryo can form skeletal muscle. The precursor myoblasts are formed first, and then the cell fusion forms multinucleated primitive muscle fibers with a nucleus in the center after the proliferation of myoblasts. The number of muscle fibers in mammals remains constant after birth development (1, 2); however, the muscle fiber diameter increases and the tenderness decreases with age in this growth stage, and various muscle fibers transform each other simultaneously (3). The different types of muscle fibers are closely related to meat color, tenderness, water-holding, juiciness, and flavor. Our previous study has identified four types of muscle fibers (Types I, IIa, IIx, and IIb) in the LT muscle of the Tibetan sheep at different ages, corresponding to the four myosin heavy chains, MyHC I, MyHC IIa, MyHC IIx, and MyHC IIb, respectively (40).

*MYH4* is a differentially expressed gene in the LT muscle of 4 months *vs*. 1.5 years group, while, there was no difference in the group of the 1.5 years *vs*. 3.5 years and 3.5 years *vs*. 6 years. *MYH1* and *MYH4* were differential expressed; however, *MYH2* and *MYH7* were not expressed in the group of 4 months *vs*. 6 years. *MYH1, MYH2, MYH4*, and *MYH7* encode MyHC IIx, MyHC IIa, MyHC IIb, and MyHC I types of muscle fibers in the mammalian skeletal muscle, respectively. Generally, the type I fibers generally contain more lipids than the type IIb fibers, whereas a high content of type IIb fiber accounts for the toughness of the meat (41). The previous studies have identified the types of muscle fibers in the skeletal muscles to transform each other with the increase of age (42). Also, AMPK is an important signaling pathway that transforms the muscle fiber types, which is mainly linked with the regulation of the energy and energy regulators of the cells (43). This study, identified the DEGs in the 4 months *vs*. 1.5 years group as the most significantly enriched (*p* < 0.05) in the AMPK signaling pathway (*p* = 0.00006), with 14 annotated DEGs including *LIPE, PCK1, IRS2, PFKFB2, LEP, EEF2*, and *ACC1*, which were closely related to cell proliferation and metabolism. The lipase E hormone sensitive (*LIPE*) is a type of lipid catabolism enzyme, with a key role in regulating the deposition of fat tissues. The genetic polymorphism of *LIPE* has been closely related to the dressing percentage of sheep (44).

*PFKFB2* is related to cell proliferation (45) and survival (46). *PCK1* was originally identified as a gluconeogenic enzyme and has been recently shown to possess protein kinase activity (47). *Leptin* (*LEP*) is a kind of adipokines produced by adipocytes or other cells. *In vitro* studies have found *LEP* to stimulate the activation and proliferation of the endothelial cells (48). The eukaryotic translation elongation factor (*EEF2*), controls the translational elongation of proteins and inhibits protein production. ACC1 is an acetyl-CoA carboxylase, providing malonyl-CoA for mitochondrial biogenesis (49). The content of mitochondria was found to be different for the different types of muscle fibers, so ACC1 might be involved in muscle fiber types switching. This study found significant enrichment of *PRKAG2, ADIPOQ, PIK3R3*, and *ACACA* in the AMPK signaling pathway in the 1.5 years *vs*. 3.5 years group (*p* < 0.05). These genes were mainly involved in the lipid metabolism pathways. *PRKAG2* participates in regulating the AMPK activity and further affects the AMPK signaling pathway. *ADIPOQ* plays an important role in fat metabolism regulation, mainly by binding to the receptors and playing a biological function by acting on the target tissues. ADIPOQ receptor 1 (AdipoR1) preferentially binds to the spherical region of *ADIPOQ*, which is expressed in the skeletal muscle cells and acts through the AMPK and mitogen activated protein kinase (MAPK) pathways (50). *ACACA* is an acetyl-Coenzyme A carboxylase α is a key rate limiting enzyme for synthesizing fatty acid, and plays an important regulatory role in fatty acid biosynthesis (51).

The genes of *SREBF1, PRKAG2, CREB5*, and *SLC2A4* were significantly enriched in the AMPK signaling pathway in the 3.5 years *vs*. 6 years group (*p* < 0.05). The sterol regulatory element binding proteins (*SREBPs*) maintain lipid homeostasis by regulating target genes (52). Solute carrier family 2 member 4 (*SLC2A4*) has been related to the contraction of the skeletal muscles (53). Fifteen DEGs including *ACACB, IRS2, IGF1R, Ppp2r2d, PCK1, LIPE, SCD*, and *FASN* were found to be significantly enriched in the 4 months *vs*. 6 years group (*p* < 0.05) in the AMPK signaling pathway. *ACACB* was possibly involved in regulating fatty acid oxidation rather than fatty acid biosynthesis, thereby affecting the milk production of dairy cows (54). The type 1 insulin-like growth factor receptor (*IGF1R*) located on the cell membrane, is activated by the insulin-like growth factor (*IGF1* or *IGF2*) and regulates the growth and differentiation of the cells, as well as the growth, development, and senescence of higher animals (55). *SCD* is stearoyl-CoA desaturase, previously identified to reduce adipogenesis in skeletal muscle upon inhibition of the *SCD* gene expression (56), while the knockout of *SCD* has been found to accelerate the accelerated fatty acid catabolism (57). *FASN* is a fatty acid synthase regulating the content of the saturated fatty acids in milk and meat (58), and further improving a human healthy diet.

In addition, the PPAR signaling pathway, glutathione metabolism, cell adhesion molecules (CAMs), insulin signaling pathway, and FoxO signaling pathway were significantly enriched in this study. The peroxisome proliferator activated receptor (PPAR) is a member of type II nuclear receptor superfamily, and a type of nuclear transcription factor activated by the ligands, which critically regulates lipid metabolism, adipogenesis, insulin sensitivity, inflammation, cell growth, and differentiation (59). There were significant enrichment of the genes *PPARA, SCD*, and *APOA1* (*p* < 0.05) in the PPAR signaling pathway. These genes are related to lipid metabolism and also play a positive effect in regulating the muscle fiber types (60). The IGF-I of the insulin signaling pathway stimulates the differentiation rate of the myoblasts and affects the expression of the myogenic regulatory factor family genes (61). The FoxO signaling pathway is critical for cell proliferation, apoptosis, autophagy and inflammation (62, 63). The possible interaction of the signaling pathways involved in muscle, fat, and connective tissue development, form a complex regulatory network that affects muscle fiber development and type composition. These signaling pathways might be involved in muscle development and transformation of the muscle fiber type.

In this study, MM.green, MM.grey60, and MM.dark-red related to the meat quality and slaughter performance were identified using WGCNA. Among these modules, *P4HA2, PLXND1, COL22A1, COL5A1, NID2*, and *Sox8* were identified as the hub gene in the interaction network of MM.green module. *FBXL4, FBXO32, TBC1D17*, and *PCMTD2* were identified as the hub gene in the MM.grey60 module. *PPARA, ADAM30, UBTFL1, EGR2*, and *KNDC1* were identified as the hub gene in the MM.dark-red module. P4HA2 is an important factor affecting hypoxia-inducible factor 1α (64), is a central gene for the Tibetan sheep to adapt to high altitude hypoxic conditions. *PLXND1* regulates the cell patterns by regulating the structure of the cytoskeleton and adhesion proteins (65). *COL22A1* encodes collagen, and knocking out the *COL22A1* gene in zebrafish can lead to muscle atrophy (66), showing that *COL22A1* is related to muscle development. The SOX family is a type of transcription factor regulating the genes related to organ development. and cell stemness and differentiation; SOX8 not only participates in the normal physiological functions but also closely affects the occurrence and development of tumors (67). The proteins FBXL4 and FBX032 are related to the cell cycle transition, apoptosis, transcription regulation, cell signal transduction, and other various physiological functions by recognizing and degrading substrate proteins (68). *TBA1D17* might be involved in mitochondrial autophagy, and the content of mitochondria in muscle cells might be related to the muscle fiber types switching. *PPAR*α regulates the expression of many proteins related to fat metabolism homeostasis (69). This study, investigated changes in the multiple genes involved in muscle growth and the difference in meat tenderness of LT muscle in Tibetan sheep at different ages. The DEGs, GO terms, KEGG signaling pathway and hub genes helped to better understand the muscle growth and development and meat quality of Tibetan sheep at different ages, which would benefit in improving the meat quality in the Tibetan sheep in the future.

## Conclusion

In conclusion, the results indicated 1.5 years as the most suitable slaughter age of Tibetan sheep. The KEGG enrichment results showed that *LIPE, LEP, ADIPOQ, SCD*, and *FASN* might be participated in the AMPK signaling pathway to regulate the muscle development and muscle fiber types transformation of Tibetan sheep, thereby effecting the meat quality. *P4HA2, COL22A1, COL5A1, FBXO32, TBC1D17*, and *Sox8* were related to the meat tenderness and protein content. *PPARA, EGR2*, and *ADAM30* were related to the IMF content. This study provides a theoretical foundation for studying the mechanism for the difference of Tibetan sheep meat quality at the molecular level and further to improve the meat quality traits.

## Data Availability Statement

The datasets presented in this study can be found in online repositories. The names of the repository/repositories and accession number(s) can be found in the article/Supplementary Material.

## Ethics Statement

The animal study was reviewed and approved by the Faculty Animal Policy and Welfare Committee of Gansu Agricultural University (Ethic approval file No. GSAU-Eth-AST-2021-001).

## Author Contributions

YW: data curation and writing—original draft. JW, XL, and GB: formal analysis, methodology, and software. JH, ZZ, BS, and FZ: investigation, validation, methodology, and software. SL: funding acquisition and writing—review and editing. YL: project administration, supervision, and writing—review and editing. All authors contributed to the article and approved the submitted version.

## Funding

This work was supported by the fund of Basic Research Creative Groups of Gansu Province (17JR5RA137), the Fuxi Young Talents Fund of Gansu Agricultural University (Gaufx-03Y04) and the Projects of Gansu Agricultural University (GSAU-ZL-2015-031/033), and the key R&D projects in Gansu Province (18YF1WA082).

## Conflict of Interest

The authors declare that the research was conducted in the absence of any commercial or financial relationships that could be construed as a potential conflict of interest.

## Publisher's Note

All claims expressed in this article are solely those of the authors and do not necessarily represent those of their affiliated organizations, or those of the publisher, the editors and the reviewers. Any product that may be evaluated in this article, or claim that may be made by its manufacturer, is not guaranteed or endorsed by the publisher.

## Supplementary Material

Supplementary Figure 1 AMPK signaling pathway.

Supplementary Table 1 The information of PCR primer used for RT-qPCR.

Supplementary Table 2 The summary of the RNA-seq data.

Supplementary Table 3 The information of DEGs of the contiguous period transcriptome comparative groups.

Supplementary Table 4 The results of GO annotation in the contiguous period transcriptome comparative groups.

Supplementary Table 5 The results of KEGG signaling pathway in the contiguous period transcriptome comparative groups.
