# Supplementary material for: Comparative Transcriptome Analysis Reveals the Mechanism Associated With Dynamic Changes in Meat Quality of the Longissimus Thoracis Muscle in Tibetan Sheep at Different Growth Stages
Source: Front Vet Sci. 2022 Jul 6;9:926725. doi: 10.3389/fvets.2022.926725 (PMC9298548; doi:10.3389/fvets.2022.926725)
Supplement: Supplementary Figure 1 — AMPK signaling pathway. [file Data_Sheet_1.docx]

**Appendices**

**Table S1 The information of PCR primer used for RT-qPCR.**

| Gene | Forward (5’→3’) | Reverse (5’→3’) |
| --- | --- | --- |
| P4HA1 | CGAGCCACCATTTCAAACCCA | AATTCGTGACACCACAGGGT |
| RPL10A | CCCTGCTCACACACAACGAA | AGAAGTTGACAGCCAGGTGA |
| ANKRD2 | CACCCATCAGAAGTTGCCCA | GATCTCCCTCCGCAGGTCTA |
| DCXR | GGTCGCAGTACTGTCAAGGC | TCCCAGTCAGCTAGGTCCAC |
| Myl6b | AGTGATGAGCTGAAGTCCCG | TTTGTCAAACACCCGAAGCC |
| HSPA8 | CGGTGATGCTGCAAAGAACC | CTTGAACCTTAGGCCTGCCA |
| MYBPH | ACCTGCAAATCCCCTTCCAG | AGAGGATGGAGTCCTGGTCC |
| CSRNP1 | GGAGGATTGATCGCGAGGAG | CTGCAGCTACAGGTCTCAGG |
| EGR1 | GCCACCACATATTCCTCCGT | TCATGTCCGAAAGCCCAGTG |
| NMRK2 | GACACCCACATCCTCATTCTGG | AGCTTCGCCTACTTCTCCTCCA |
| ASB2 | CCTGCTTCTCGTGCCTGTAT | GTTGCCCACGTAGTCCAAGA |
| SESN1 | TGAGATGGGATGGACTGTGC | CGCATTTGAAAGGCCGTCTG |
| HYAL2 | ATTTGTCAAGGCGTTTCGGC | ACAGCTGGTCATTTCGGGAG |
| CRYAB | GATATCGCCATCCACCACCC | GGGCTCAGGGAAGTAGAAGC |
| TFRC | CAAAGTTTCTGCCAGTCCGC | GATCCAGTTGCTGTCCCGAT |
| FBXO32 | AGGCTGGACTTCTCAACTGC | CCACTCAGGGATGTGAGCTG |
| TXNIP | ATCCCCAAAGCTGCCATTGT | TCTTCTGCACTCGAAGGCTC |
| GAPDH | CTTCTGCTGACGCTCCCA | AAGTCCCTCCACGATGCC |

**Table S2. The summary of the RNA-Seq data**

| Sample | Average raw  reads | Average clean  reads | Average remaining  clean reads | Average  mapped reads | Average  unique reads | Average  multiple reads |
| --- | --- | --- | --- | --- | --- | --- |
| 4 m | 91,167,071 | 90,738,055 | 90,373,003 | 88,176,017  (97.57%) | 81,146,261  (92.03%) | 7,029,756  (7.97%) |
| 1.5 y | 95,150,638 | 94,864,498 | 94,163,662 | 88,912,904  (94.42%) | 81,647,895  (91.83%) | 7,265,008  (8.17%) |
| 3.5 y | 90,313,673 | 89,970,024 | 89,518,448 | 82,392,489  (92.04%) | 75,993,221  (92.23%) | 6399268  (7.77%) |
| 6.0 y | 87,472,429 | 87,228,506 | 86,739,090 | 80,924,685  (93.30%) | 74,245,776  (91.75%) | 6678909  (8.25%) |

Figure S1. AMPK signaling pathway. The white square represents a gene or protein, the red square is the up-regulated genes in the pathway, the green square is the down-regulated genes in the pathway. (A-C) AMPK signaling pathway in the 1.5 y-vs-3.5 y group, 3.5 y-vs-6 y group, 4 m-vs-6 y group, respectively.
